# Acclimatization Effects of *Senecio nutans* Administration in Female Rats Exposed to Acute Hypobaric Hypoxia

**DOI:** 10.3390/ijms27136080

**Published:** 2026-07-07

**Authors:** Karen Flores, Karem Arriaza, Eduardo Pena, Isaac Cortes, Maite Villalobos, Samia El Alam

**Affiliations:** 1 High Altitude Medicine Research Center (CEIMA), Arturo Prat University, Iquique 1110939, Chile; kfloresu@unap.cl (K.F.); karriaza@unap.cl (K.A.); 2 Mathematic Department, Engineer Faculty, Atacama University, Copiapó 1530000, Chile; isaac.cortes@uda.cl; 3 Center for Applied Research and Innovation in Biological Sciences, Faculty of Renewable Natural Resources, Arturo Prat University, Huayquique Campus, Iquique 1100000, Chile; maitevillalobos12@outlook.es

**Keywords:** acute hypobaric hypoxia, *Senecio nutans*, acclimatization, inflammation, lung injury

## Abstract

Exposure to high altitudes for hours or days is defined as acute hypobaric hypoxia (AHH) condition, which rapidly engages adjustments such as signaling pathways involving inflammation, immune modulation and oxidative stress, whose dysregulation has been described as contributing to the pathophysiology of high-altitude illnesses, due to insufficient acclimatization, such as developing acute mountain sickness (AMS). Given its traditional high-altitude use and bioactive properties, *Senecio nutans* (*S. nutans*) extract, or chachacoma (CH), has emerged as a potential therapeutic strategy to mitigate high-altitude related pathobiology. The aim of this study was to evaluate the effects of *S. nutans* on acclimatization, regarding the status of oxidative stress, inflammation, immune and symptoms associated with AMS in an animal model exposed to AHH. Twenty-eight female Wistar rats (≈3 months old) were randomly allocated into four experimental groups (n = 7 each): normobaric normoxia (NX), normobaric normoxia plus *S. nutans* administration (NX+CH), acute hypobaric hypoxia (AHH; 48 h exposure), and acute hypobaric hypoxia plus *S. nutans* administration (AHH+CH). *S. nutans* was administered subcutaneously at a dose of 80 mg/kg, one hour prior to hypoxic exposure. Outcomes included body weight, food intake, hematological parameters, lung histopathology, pulmonary mRNA expression of HIF-1α, NF-κB, TNF-α, IL-1β, IL-6, and VEGF, and lipid peroxidation in lung tissue assessed by malondialdehyde (MDA) levels. After 48 h of AHH, animals exhibited a decrease in body weight and food intake, increase in hematocrit level and total leukocytes, as well as lung injury characterized by thickening of alveolar walls and inflammatory infiltrates. In addition, AHH induced an increase in pulmonary IL-6 and IL-1β mRNA expression. In contrast, *S. nutans* administration partially attenuated hypoxia-induced body weight loss, mitigated the rise in hematocrit levels, and reduced lung damage, while returning total leukocyte counts to control levels. Notably, *S. nutans* also decreased the hypoxia-induced overexpression of IL-6 and IL-1β. Regarding lipid peroxidation, no significant differences were observed among groups. These findings suggest that *S. nutans* exerts a protective effect against acute hypobaric hypoxia by attenuating inflammatory responses and preserving pulmonary structure, thereby supporting its potential as a preventive strategy to mitigate early pathophysiological alterations associated with high-altitude exposure.

## 1. Introduction

Exposure to geographical altitude over 2500 m above sea level (m.a.s.l) produces a decrease in partial pressure of oxygen, leading to hypobaric hypoxia condition, which triggers a series of physiological responses that generally enable individuals to tolerate and acclimatize to low-oxygen conditions [1,2]. However, in some people, these acclimatization mechanisms become dysregulated, leading to exaggerated responses that cause altitude-related illnesses [1,2]. The first hours or days of ascension to high altitudes considered as acute hypobaric hypoxia (AHH) exposure produce an initial physiological response comprising hyperventilation triggered by the hypoxic ventilatory response, hemoconcentration due to an increase in red blood cells to enhance the transport of oxygen to the tissues and enhanced diuresis, and an increase in heart rate and cardiac output as a result of sympathetic activation in hypoxia at altitude [3]. An adequate ventilatory response is critical for proper acclimatization; appropriate acclimatization to hypoxia can minimize or even prevent those illnesses. Ventilatory acclimatization to hypoxia supports the recovery of the initially reduced alveolar (PAO_2_) and arterial (PaO_2_) partial pressures of oxygen and arterial oxygen saturation (SaO_2_) [4].

The people who are not acclimatized develop acute mountain sickness (AMS) within the first 6 to 12 h of acute high-altitude exposure, one of the main and the most frequently observed altitude illnesses, characterized by symptoms such as headache, gastrointestinal disturbances (loss of appetite, nausea, and vomiting), fatigue, weakness, and dizziness [1,4]. In animal models, specifically murine, reduced food intake and weight loss have been widely described as a characteristic physiological response to hypobaric hypoxia and have been associated with AMS [5]. At molecular level, AHH activates early signaling pathways that involve inflammatory mediators, immune dysregulation, and increased oxidative stress mechanisms that have been strongly associated with the development of AMS [6,7,8]. In susceptible individuals, the overactivation of these pathways results in maladaptive responses that aggravate AMS symptoms [6,9]. Pro-inflammatory cytokines such as interleukin IL-6 and IL-1β are consistently elevated under hypobaric hypoxic conditions [10,11,12]. Concurrently, an increase in reactive oxygen species has been documented, with both oxidative stress and inflammation playing central roles in the onset and progression of AMS [9].

In parallel, several studies have reported an elevation in white blood cell counts during exposure to hypobaric environments [13,14]. AHH-related illnesses, such as AMS and high-altitude pulmonary edema (HAPE), are characterized by an inflammatory response, evidenced by elevated levels of pro-inflammatory cytokines and leukocytes in both alveolar lavage fluid and systemic circulation [10,15]. These findings are consistent with results from animal studies, in which increased leukocyte counts have been observed following exposure to AHH [16].

The primary pharmacological treatment for alleviating AMS symptoms is oral acetazolamide; however, its adverse effects often lead some individuals to discontinue or avoid its use when symptoms persist [17]. Current research is increasingly exploring alternative therapeutic options, including natural compounds such as antioxidants, polyphenols, and plant-derived extracts [18]. *S. nutans* (Sch. Bip) is a highly complex and cosmopolitan genus within the Asteraceae family, comprising over 1000 species, depending on taxonomic circumscription [19]. It includes herbaceous and shrubby forms, and many species occur in high-Andean environments, typically between 3500 and 5000 m.a.s.l. In South America, Chile and Argentina host the greatest regional diversity, each with around 260–300 species, according to classical floristic treatments [20,21].

In northern Chile, *S. nutans*, commonly known as “chachacoma”, is distributed across the high-altitude regions of Arica y Parinacota, Tarapacá, and Antofagasta, and is widely used by Andean communities [22]. It is commonly consumed as an infusion by travelers and workers to alleviate symptoms of AMS [23]. In addition to its traditional use, *S. nutans* has been reported to exhibit antioxidant and anticancer activities [22,24]. However, its molecular and physiological effects under hypobaric hypoxia remain poorly understood. Previous studies (not conducted at high altitude) have shown an antihypertensive effect in rats, associated with reduced heart rate, cardiac contractility, and sarco/endoplasmic reticulum Ca^2+^-ATPase activity [25], suggesting a potential cardioprotective role.

Despite its cultural relevance, the pharmacological characterization of *S. nutans* in the context of high-altitude conditions remains limited, particularly for populations from the Tarapacá region. This is noteworthy given that the high-Andean ecosystems of Tarapacá exhibit distinctive environmental stressors, including exceptionally high solar radiation, extreme aridity, and elevated soil and water salinity, all of which may influence the plant’s biological activity [26]. Therefore, this study aims to evaluate, for the first time, the effects of *S. nutans* on acclimatization to AHH by assessing physiological, hematological, inflammatory, and hypoxia-related responses, along with pulmonary alterations in an animal model.

## 2. Results

### 2.1. Body Weight and Food Intake

We first conducted an AHH experiment to evaluate the *S. nutans* protective effects. After 48 h of AHH, body weight (BW48) of the animals exposed to AHH and AHH+CH decreased compared to baseline body weight (BW0). However, only the decrease in the AHH group was significantly different to the normoxic control (NX) group (Table 1). Food intake was also reduced in both the AHH and AHH+CH groups after 48 h of exposure (Table 1), with the lowest intake observed in the AHH group. Although no significant differences were detected between the two groups, this reduction aligns with the body weight loss observed in both experimental conditions.

### 2.2. Hematological Parameters

**Figure 1:**
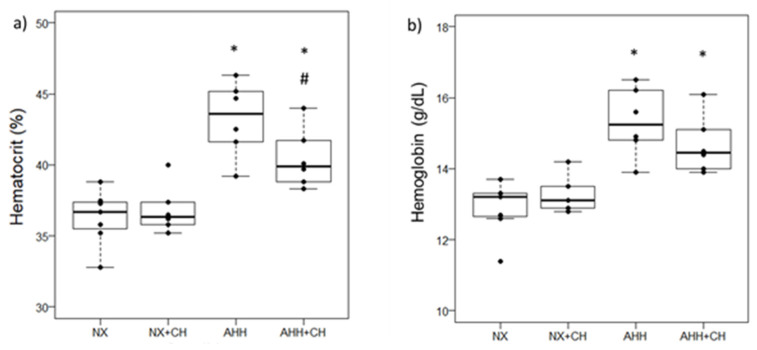
Blood sample analysis of (**a**) hematocrit percentage (%) and (**b**) hemoglobin (g/dL) of rats exposed to normoxia (NX), normoxia plus *S. nutans* (NX+CH), acute hypobaric hypoxia (AHH), acute hypobaric hypoxia plus *S.nutans* (AHH+CH) after 48 h. Boxes indicate the interquartile range and the central line represents the median. * *p* < 0.05 vs. NX group; # *p* < 0.05 vs. AHH group.

After 48 h of exposure to AHH, blood sample analysis showed an increase in Hct%, Hb and RBC in both AHH and AHH+CH groups compared to the normoxic control (NX) group (Table 2). Notably, Hct% was lower in the *S. nutans*-treated hypoxic group (AHH+CH) than in the AHH group (*p* = 0.046; Figure 1a), whereas Hb levels were not affected by *S. nutans* administration (Figure 1b). The level of MCHC was higher in both groups with *S. nutans* administration (NX+CH and AHH+CH) compared to the NX group, and MCHC values in AHH+CH were also higher than those observed in the AHH group (Table 2). MCV was increased in the AHH group compared with the NX+CH group. Regarding total leukocyte counts, rats exposed to AHH showed higher levels compared to both normoxic groups (NX and NX+CH), whereas *S. nutans* administration in hypoxic rats maintained leukocyte levels comparable to those of the normoxic control group (Table 2). In addition, the monocyte percentage was higher in NX+CH, AHH and AHH+CH groups compared with the NX group.

### 2.3. Lung Histopathological Analysis

**Figure 2:**
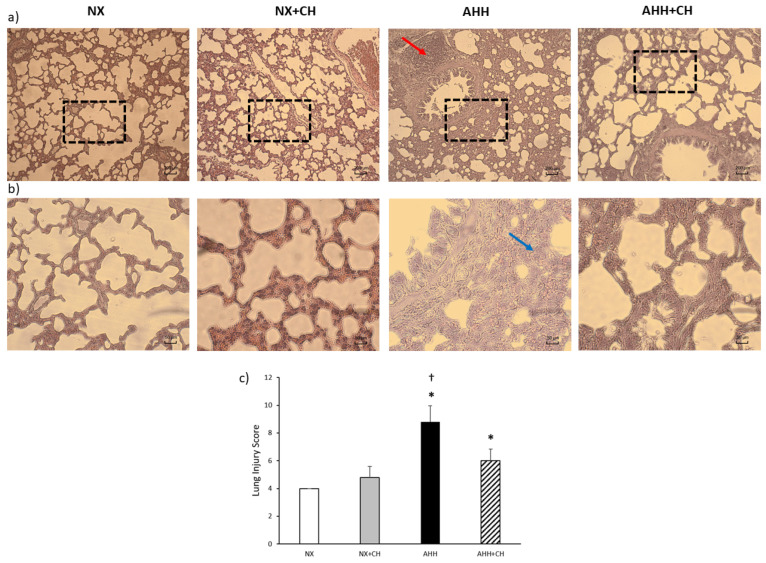
Representative image of haematoxylin and eosin-stained sections of rat lung tissue (**a**) at low magnification (10×; scale 200 μm). (**b**) High-magnification images (40×; scale 50 μm) highlighting alveolar architecture. The red arrow indicates areas of inflammatory cell infiltration, and the blue arrow indicates alveolar wall thickening. (**c**) Lung injury score (n = 5 per group). * *p* < 0.05 vs. NX; † *p* < 0.05 vs. NX+CH.

Results of hematoxylin and eosin (H&E) staining in lung tissue showed markedly histopathological changes in the alveoli analysis among the groups (Figure 2a,b). Compared with the NX control group, the AHH group exhibited pronounced histopathological alterations characterized by alveolar septa with marked thickening and congestion, blood vessels showing mild hyperemia, predominantly mononuclear inflammatory infiltrate and hypertrophy of peribronchial vessels. In the AHH+CH group, these changes were attenuated by *S. nutans* administration, with a reduction in septal thickening and congestion, decreased inflammatory infiltrate and peribronchial vascular hypertrophy. In agreement with these observations, the lung injury score was significantly increased in both AHH and AHH+CH groups compared to NX. Although the AHH+CH group showed a reduction in lung injury score relative to AHH, this decrease did not reach statistical significance. Moreover, no significant differences were observed between the AHH+CH and NX+CH groups (Figure 2c).

### 2.4. Lipid Peroxidation in Lung Tissue

**Figure 3:**
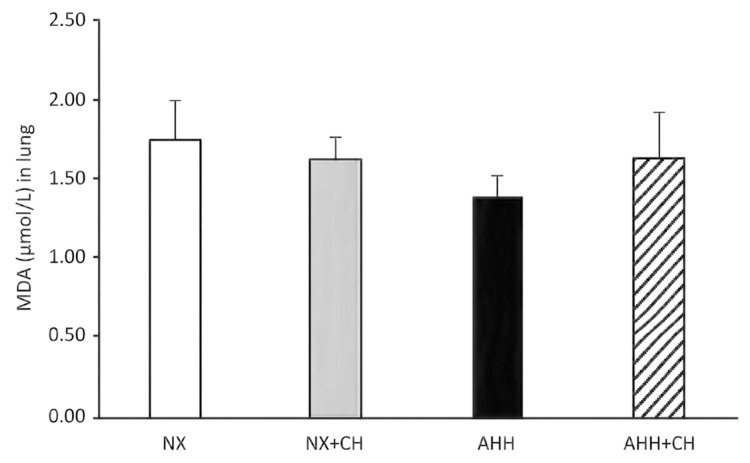
Malondialdehyde (MDA) concentration (μmol/L) in lung tissue of rats exposed to normoxia (NX), normoxia plus *S. nutans* (NX+CH), acute hypobaric hypoxia (AHH), acute hypobaric hypoxia plus *S. nutans* (AHH+CH) after 48 h. Data are expressed as mean ± SEM. ns (*p* > 0.05).

Assessment of malondialdehyde (MDA) levels in lung tissue revealed no significant differences among the experimental groups (*p* > 0.05). These findings indicate that lipid peroxidation, as evaluated by this biomarker, was not significantly modified under the experimental conditions studied (Figure 3).

### 2.5. Relative mRNA Expression in Lung Tissue

**Figure 4:**
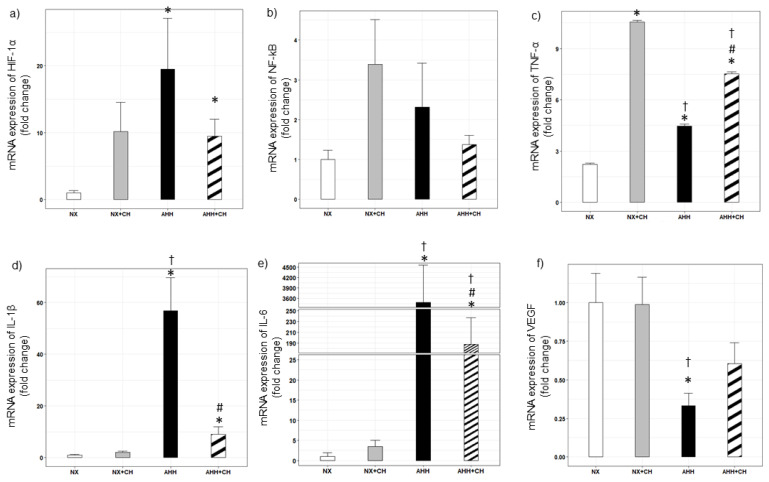
Relative mRNA expression (fold change) of HIF-1α, NF-κB, TNF-α, IL-1β, IL-6, and VEGF (**a**–**f**) in lung tissue from rats exposed to normoxia (NX), normoxia plus *S. nutans* (NX+CH), acute hypobaric hypoxia (AHH), and acute hypobaric hypoxia plus *S. nutans* (AHH+CH) for 48 h. Data are expressed as mean ± SEM. * *p* < 0.01 vs. NX; # *p* < 0.01 vs. AHH; † *p* < 0.01 vs. NX+CH.

Quantitative PCR analysis of lung tissue demonstrated a significant increase in HIF-1α expression in both hypoxic groups (AHH and AHH+CH) compared to the normoxic control (NX) group (*p* < 0.01; Figure 4a). In contrast, no significant differences in NF-κB mRNA expression were observed among the experimental groups (*p* > 0.01; Figure 4b). TNF-α mRNA expression was significantly increased in the NX+CH, AHH, and AHH+CH groups compared with the control (NX) group (*p* < 0.01; Figure 4c). Consistently, IL-1β expression was significantly upregulated under hypoxic conditions, with the AHH group showing a marked increase in IL-1β mRNA levels compared with the NX group (*p* = 0.004; Figure 4d). Notably, administration of *S. nutans* significantly reduced IL-1β expression in the AHH+CH group compared with the AHH group (*p* = 0.002). No significant differences were observed between the NX and NX+CH groups. Similarly, IL-6 relative expression was significantly elevated in the hypoxia groups (AHH and AHH+CH) compared with the normoxic control (NX) group (*p* < 0.01). However, *S. nutans* administration significantly attenuated IL-6 expression in the AHH+CH group compared to AHH group (*p* = 0.004; Figure 4e). Regarding VEGF expression, a significant decrease was observed in the AHH group compared with the normoxic groups (*p* < 0.01; Figure 4f).

## 3. Discussion

To our knowledge, this is the first study to evaluate the effects of *S. nutans* in a rat model of AHH. This native plant from northern Chile has long been used in traditional medicine to alleviate symptoms of acute mountain sickness (AMS). Our main findings indicate that *S. nutans* administration under acute hypobaric hypoxia (1) ameliorates body weight loss; (2) prevents excessive increase in Hct%; (3) attenuates alveolar wall thickening and inflammatory infiltration; (4) returns total leukocyte count to control levels; (5) decreases the hypoxia-induced overexpression of IL-1β and IL-6 in lung tissue.

Exposure to AHH induced significant reductions in body weight and food intake, responses widely recognized as early markers of hypoxia-related stress and AMS development [27,28]. Notably, *S. nutans* partially attenuated these effects, suggesting improved physiological adaptation and reduced systemic stress produced by hypoxia exposure. Although its metabolic effects remain poorly characterized, this attenuation may involve modulation of hypoxia-sensitive pathways such as HIF-1α signaling and neuroendocrine responses, both central to energy balance under hypoxia [29,30]. This is particularly relevant, as appetite suppression contributes to fatigue and diminished performance at high altitude.

Hematological adaptations are essential for increasing oxygen-carrying capacity during hypoxia; however, excessive erythropoiesis can impair microcirculatory flow and reflect poor acclimatization [31,32]. In the present study, AHH significantly elevated hematocrit, hemoglobin, and erythrocyte counts, consistent with previous evidence [33,34]. Importantly, *S. nutans* attenuated the increase in hematocrit, suggesting a regulatory effect on early erythropoietic or hemoconcentration processes. This observation aligns with reports describing the capacity of bioactive compounds to preserve hematological parameters under acute hypobaric hypoxia [33,34]. In addition, the known vasodilatory and hypotensive properties of *S. nutans* [35,36] may contribute to improved hemodynamic regulation and reduced hypoxia-induced vasoconstriction.

Histopathological evaluation of lung tissue revealed structural alterations associated with AHH exposure. In the present model, AHH induced pronounced alveolar alterations, characterized by marked alveolar wall thickening and inflammatory cell infiltration. These findings are consistent with previous reports in rats exposed to hypobaric hypoxia for 24, 48, and 72 h, in which the most severe alveolar injury was observed at 48 h and involved extensive inflammatory infiltration, diffuse red blood cell extravasation, and pronounced endothelial edema within alveolar capillaries [9]. Treatment with *S. nutans* tends to attenuate these histopathological changes including a reduction in septal thickening and inflammatory infiltration. Although these findings suggest a potential protective effect on alveolar architecture, the lack of a significant reduction in lung injury score indicates that this effect may be modest under the present experimental conditions. In this context, *S. nutans* may contribute to the preservation of the alveolar–capillary barrier, possibly through anti-inflammatory and endothelial-modulatory mechanisms [37,38]; however, further studies are required to confirm these effects and elucidate the underlying pathways.

The inflammatory response represents a central component of hypoxia-induced pathology. In agreement with prior human and animal studies, AHH increased circulating leukocytes and promoted inflammatory infiltration [13,14,16]. In our study, *S. nutans* restored total leukocyte levels toward normoxic values, indicating a systemic immunomodulatory effect. At the lung molecular level, hypoxia significantly upregulated IL-6 and IL-1β expression, consistent with established evidence [9,39], while *S. nutans* attenuated this response, reinforcing its anti-inflammatory potential. Interestingly, TNF-α expression increased not only under hypoxia but also in normoxic animals treated with *S. nutans*, suggesting a basal stimulatory effect on inflammatory signaling. This pattern may reflect a hormetic response, whereby mild activation of stress pathways enhances adaptive capacity [40,41]. Furthermore, it has been reported that TNF-α expression can be regulated through the activation of signaling pathways distinct from those involved with other inflammatory factors. In this regard, certain phytochemicals can preferentially activate the early stages of the TNF-α-mediated inflammatory response, significantly increasing its transcription, which suggests potential immunostimulatory properties [42]. Moreover, Zhu et al. observed that TNF-α expression increases to promote immunostimulation following the administration of flavonoids from *Senecio scandens* [43]; this effect could be reflected in the increased monocyte count observed in the normoxic group treated with *S. nutans* in the present study. Notably, plant-derived secondary metabolites such as flavonoids exhibit broad immunomodulatory potential, including the activation of macrophage phagocytic functions [44].

HIF-1α, the master regulator of cellular adaptation to hypoxia, was markedly upregulated after AHH, confirming activation of canonical oxygen-sensing pathways [28,45]. Interestingly, *S. nutans* administration partially attenuated this increase, although expression levels remained elevated compared with normoxic conditions. A similar modulation pattern was observed for NF-κB expression, suggesting that *S. nutans* may not completely suppress hypoxia signaling, but rather may limit excessive or maladaptive activation while preserving adaptive responses. This interpretation is particularly relevant considering the functional crosstalk between HIF-1α and NF-κB pathways during hypoxia-induced inflammation and pulmonary injury [45,46]. Furthermore, the mild increase observed under normoxic conditions in animals treated with *S. nutans* may support the hypothesis of a basal adaptive priming effect, potentially contributing to improved tolerance to subsequent hypoxic stress.

In contrast to the marked anti-inflammatory effects, lipid peroxidation (MDA levels) did not differ among groups. This suggests that oxidative stress, at least at the level of lipid peroxidation, may not be a dominant mechanism at this early stage (48 h) of hypoxic exposure, or that antioxidant defenses were sufficient to maintain redox balance. Previous studies report variable oxidative responses depending on exposure duration and tissue type [9], indicating complex regulation. Therefore, the protective effects of *S. nutans* in this model appear to be primarily mediated by anti-inflammatory rather than direct antioxidant mechanisms.

Finally, VEGF expression was significantly reduced under AHH, indicating a potentially impaired adaptive response. As VEGF is essential for maintaining pulmonary vascular integrity and angiogenesis during hypoxia, its downregulation may reflect endothelial dysfunction and disruption of the alveolar–capillary barrier. This paradoxical decrease has been associated with dysregulated HIF-1α signaling, inflammation, or oxidative imbalance under severe hypoxic stress [46,47] and may contribute to compromised acclimatization and structural lung alterations. Collectively, these findings indicate that *S. nutans* provides multi-target protection against acute hypobaric hypoxia by modulating inflammation, preserving lung architecture, and regulating hematological responses. This integrative effect positions *S. nutans* as a promising candidate for mitigating early hypoxia-associated pathophysiological processes.

The results of this study have relevant translational implications for the prevention and management of AMS and other high-altitude-related conditions. AMS remains a common and sometimes debilitating condition affecting individuals who ascend rapidly to altitudes above 2500 m, including tourists, military personnel, and high-altitude workers [1]. In addition, studies demonstrated the susceptibility to developing AMS in women due to hormone factors [48,49]. Current pharmacological treatments, such as acetazolamide, are effective but often associated with adverse effects that limit adherence [15]. 

In this context, *S. nutans* emerges as a promising complementary or alternative strategy. Its demonstrated ability to attenuate key features of hypoxia-induced pathology—such as inflammation, hematological imbalance, and lung injury—suggests that it may contribute to improving acclimatization and reducing symptom severity. Furthermore, its potential vasodilatory and cardioprotective effects [22,34] could be particularly beneficial in preventing complications such as HAPE and cardiovascular strain at altitude. 

However, translation to clinical practice requires caution. Further studies are needed to determine optimal dosing, administration routes, safety profiles, and efficacy in humans. Clinical trials in high-altitude settings will be essential to validate these findings and to position *S. nutans* within evidence-based therapeutic strategies for AMS. Overall, this study provides a strong experimental foundation supporting the integration of traditional Andean phytotherapy into modern high-altitude medicine, with *S. nutans* representing a particularly promising candidate.

This study has several limitations that should be acknowledged. Although significant anti-inflammatory and histological effects were observed, the molecular mechanisms underlying the action of *S. nutans* were not fully characterized, particularly those related to hypoxia-inducible signaling pathways and endothelial function. In addition, the assessment of oxidative stress was limited to lipid peroxidation (MDA), which may not adequately reflect the overall redox status; therefore, the inclusion of additional oxidative and antioxidant markers would provide a more comprehensive evaluation. Furthermore, the use of a single dose and route of administration restricts conclusions regarding dose–response relationships and pharmacokinetic properties. Finally, as this study was conducted in an animal model, extrapolation of the findings to humans should be approached with caution, and further clinical studies are needed to confirm its safety and efficacy in high-altitude populations.

## 4. Materials and Methods

### 4.1. Plant Material

The branches, leaves, and inflorescences of *S. nutans* Sch. Bip. (chachacoma) were collected in the Tarapacá Region, north of Chile, near Chusmiza (19°38′36.66″ S–69° 5′50.71″ O; 3.900–4.200 m). *S. nutans* has been previously characterized through UHPLC-based analysis and reported by Palacios et al. (2023), who identified a diverse phytochemical profile mainly composed of acetophenone derivatives (e.g., dihydroeuparin and 4 hydroxy 3 (3 methyl 2 butenyl) acetophenone), along with phenolic acids such as caffeic, chlorogenic and coumaric acids, flavonoids derived from quercetin and myricetin, and additional compounds including organic acids, amino acids, oxylipins, and coumarins, totaling more than fifty tentatively characterized metabolites [25].

### 4.2. Chemical and Reagents

All the solvents (ethanol), of analytical grade (99.5%), and reagents (paraformaldehyde, hematoxylin and eosin, MDA, trichloroacetic acid, thiobarbituric acid) were purchased from Sigma-Aldrich (St. Louis, MO, USA). Grade 1 distilled (ultrapure) water was used as the solvent and for the preparation of all solutions.

### 4.3. Extract Preparation

The plant material was spread and dried in the shade at room temperature, and then with the help of a mechanical mill it was finely ground. A 2.0 kg mass of the dried, powdered plant material was macerated in a glass beaker with 4 L of an EtOH:H_2_O (1:1) mixture for 72 h. Then, the solvent was removed under reduced pressure using a DRAGONLAB RE-100 rotary evaporator (Dragon Lab, Beijing, China) at 40 °C and 120 rpm. This procedure was repeated several times, until the final solution was colorless. The concentrate obtained was freeze-dried using a 4.5 FreeZone, Labconco lyophilizer (Labconco Corp., Kansas City, MO, USA). The lyophilized hydroalcoholic extract was stored at 4 °C until use.

### 4.4. Animal Model and Study Design

Twenty-eight female Wistar rats (±3 months) obtained from the High-Altitude Medicine Research Center, Arturo Prat University, Iquique, Chile, were used in this study. All procedures and protocols involving the animals were approved by the Research Ethics Committee of Tarapacá University (N° 21/2024). Furthermore, this study was carried out in accordance with the ethical standards for the management of experimental animals (Chilean Law 20.380, Art 7, 3 October 2009). The rats were housed in individual cages at a temperature of 22 ± 2 °C on a 12 h light/12 h dark cycle; the feeding consisted of 40 g/day of food containing 22.0% crude protein, 5.0% crude fiber, 9.0% ash, and 12% moisture (5POO^®^, LabDiet, Prolab RMH3000, Richmond, IN, USA) and water was provided ad libitum. No physical exercise was performed; however, movement within the cage was not restricted.

The rats were randomly divided into 4 experimental groups (n = 7): normobaric normoxia (NX) group, which was used as a control (sea level) group; the normobaric normoxia plus *S. nutans* administration (NX+CH) group; the acute hypobaric hypoxia (AHH) group, which was exposed for 48 h under hypobaric hypoxia; and the AHH plus *S. nutans* administration (AHH+CH) group, which was treated with *S. nutans* one hour before exposure to hypobaric hypoxia. The dose of *S. nutans* was 80 mg/kg of body weight, selected on the basis of previously published dose–response studies [25], and it was administered through subcutaneous injection in the interscapular area before exposure to hypobaric hypoxia. For NX and AHH groups, saline solution was administered as a placebo.

Hypobaric hypoxia was achieved with the use of a hypobaric chamber at 428 Torr, which is equivalent to a pressure at an approximately altitude of 4600 m. The internal air flow in the chamber was 3.14 L/min, and the humidity was between 21 and 30%. The time necessary to reach the final pressure was approximately 30 min. The rats in the NX and NX+CH group were maintained under the same environmental conditions but without hypobaric hypoxia exposure.

### 4.5. Body Weight and Hematological Measures

At the beginning (0 h) and end (48 h) of the exposure period, the body weights (BWs; g) of the rats were measured via an electronic scale (Acclab V-1200^®^, Lake Country, IL, USA). After 48 h of exposure, the rats were anesthetized with ketamine (90 mg/kg body weight) and subsequently sacrificed by fatal thoracotomy, and blood and lung samples were obtained. The blood samples were obtained in EDTA vials after fatal thoracotomy via puncture of the inferior vena cava. Hematocrit (Hct, %), hemoglobin (Hb, g/dL), mean corpuscular volume (MCV; fL), mean corpuscular hemoglobin concentration (MCHC; g/dL), red blood cells (RBC, M/uL) and leukocyte count were measured.

### 4.6. Lung Histopathology

The right upper lobe of the lung was dissected and fixed in 4% paraformaldehyde for 4 weeks. Following dehydration treatment using a graded ethanol series, the tissues were embedded in paraffin and sectioned into 3 μm thickness sections. The tissue sections were stained with hematoxylin and eosin (H&E) for histopathological examination under a light microscope, focusing on assessing the thickness of alveolar walls and the extent of inflammatory infiltration. To ensure greater clarity, robustness, and specificity in the histopathological assessment, two experienced histologists independently evaluated all tissue samples in a blinded manner. Histopathological alterations were scored using a semi-quantitative system adapted from previously described methods [50,51]. Four parameters were evaluated: (i) inflammatory cell infiltration, (ii) alveolar septal thickening, (iii) vascular alterations (including congestion and hypertrophy), and (iv) architectural changes (emphysematous alterations). Each parameter was scored on a scale from 0 to 4 based on the percentage of affected area: 0, no injury; 1, ≤25%; 2, 26–50%; 3, 51–75%; and 4, ≥76%.

### 4.7. Lipid Peroxidation

Lipid peroxidation was evaluated in lung tissues by determining the malondialdehyde (MDA) concentration (μmol/L) via a colorimetric assay. First, 60 mg of lung tissue was homogenized in 600 μL of RIPA buffer (50 mM Tris-HCl, 1% Triton Pak^®^; Brinton, IL, USA) at 4 °C. Then, 100 μL of the sample was mixed with 200 μL of 10% trichloroacetic acid on ice for 30 min. The mixture was subsequently centrifuged at 4000 rpm for 15 min at 4 °C. Two hundred microliters of the supernatant was subsequently mixed with 200 μL of 0.67% thiobarbituric acid and incubated in a bath at 100 °C for 1 h. Finally, with the use of a spectrophotometer (Thermo Electron Corporation^®^, Madison, WI, USA), the absorbance was measured at 532 nm.

### 4.8. Quantitative PCR Analysis

Total RNA was isolated from lung tissue samples (50 mg) using RNA-Solv Reagent (Omega Bio-tek, Norcross, GA, USA) following the manufacturer’s instructions. This reagent is based on the phenol–chloroform extraction method described by [52]. The total RNA was reverse transcribed into cDNA using the AffinityScript™ QPCR cDNA Synthesis Kit (Agilent Technologies, Santa Clara, CA, USA), and analyzed using the PowerUp SYBR Green Master Mix kit (Applied Biosystems, Foster City, CA, USA) with the following cycling conditions: 10 min of denaturation at 95 °C, followed by 40 cycles of 10 s at 95 °C and 60 s at 60 °C. Data were normalized to β-actin and GAPDH, and relative expression was calculated using the 2^−ΔΔCt^ method. Primers were provided by Integrated DNA Technologies, Inc. (Coralville, IA, USA). The contents of primer sequences are shown in Table 3.

### 4.9. Statistical Analysis

Mean, standard deviation (SD) and standard error of the mean (SEM) were calculated for each variable across the different experimental conditions (NX, NX+CH, AHH, and AHH+CH). Between-group comparisons were performed using the non-parametric Wilcoxon rank-sum test (Mann–Whitney test), without assuming normality. Pairwise comparisons between experimental conditions were conducted using two-sided tests, and in specific cases, one-sided tests were applied based on a priori hypotheses. Within-group changes between baseline (time 0) and post-intervention measurements (48 h) were assessed using the Wilcoxon signed-rank test for paired samples. The significance level was set at α = 0.05 for all analyses, except for qPCR data, for which significance was defined as *p* < 0.01. All analyses were performed using R software (V4.4.0) [53].

## 5. Conclusions

In summary, this study provides evidence that *S. nutans* exerts protective effects against acute hypobaric hypoxia in a rat model, primarily through modulation of inflammatory and physiological responses. Treatment attenuated weight loss, hematological alterations, and pulmonary inflammation. The downregulation of IL-6 and IL-1β, together with normalization of leukocyte counts, supports a predominantly anti-inflammatory mechanism of action. The partial preservation of lung architecture further suggests a role in maintaining alveolar–capillary integrity under hypoxic stress, a key factor in the development of altitude-related pathologies. Collectively, these results support a preventive role for *S. nutans* as a natural agent capable of mitigating early pathophysiological responses to hypobaric hypoxia. Further mechanistic studies and clinical trials are needed to confirm its efficacy and safety in humans and to assess its potential integration into preventive and therapeutic strategies for high-altitude illnesses.

## Figures and Tables

**Table 1:** Body weight (BW) and food intake of study groups at baseline (0 h) and after 48 h of exposure.

Variables	NX	NX+CH	AHH	AHH+CH
BW0 (g)	295.14 ± 20.96	301.43 ± 11.22	291.71 ± 18.71	299.33 ± 22.90
BW48 (g)	300.29 ± 26.22	306.86 ± 11.63 ^b^	267.86 ± 22.04 ^abc^	279.67 ± 20.39 ^bc^
Food intake (%)	47.71 ± 11.07	43.43 ± 6.95	42.43 ± 4.83	42.17 ± 5.85
Food intake 48 (%)	48.57 ± 4.58	42.86 ± 6.36 ^a^	18.14 ± 10.04 ^abc^	22.50 ± 7.66 ^abc^

Values are means ± SD. NX: normoxia; NX+CH: normoxia plus *S. nutans*; AHH: acute hypobaric hypoxia; AHH+CH: acute hypobaric hypoxia plus *S. nutans*; body weight after 48 h of AHH (BW48); baseline body weight (BW0). ^a^ *p* < 0.05 vs. NX, ^b^ *p* < 0.05 vs. 0 h, ^c^ *p* < 0.05 vs. NX+CH.

**Table 2:** Hematological parameters of the study groups after 48 h of exposure.

Variables	NX	NX+CH	AHH	AHH+CH
Hemoglobin (g/dL)	12.89 ± 0.76	13.27 ± 0.52	15.32 ± 0.97 ^ac^	14.67 ± 0.82 ^ac^
Hematocrit (%)	36.30 ± 1.94	36.85 ± 1.71	43.25 ± 2.64 ^ac^	40.43 ± 2.11 ^abc^
MCV (fL)	59.06 ± 1.28	58.20 ± 1.21	60.47 ± 1.53 ^c^	58.65 ± 1.67
MCHC (g/dL)	35.49 ± 0.47	36.05 ± 0.33 ^a^	32.92 ± 5.94 ^c^	36.25 ± 0.28 ^ab^
RBC (M/µL)	6.13 ± 0.36	6.28 ± 0.19	7.42 ± 0.76 ^ac^	6.85 ± 0.58 ^ac^
Total leukocytes (10^3^/μL)	5.56 ± 2.31	5.07 ± 1.87	8.35 ± 1.32 ^ac^	5.17 ± 1.83 ^b^
Lymphocytes (%)	50.29 ± 35.13	66.83 ± 22.51	63.33 ± 20.54	56.50 ± 24.13
Monocytes (%)	4.29 ± 1.25	7 ± 2.45 ^a^	6.33 ± 0.82 ^a^	7 ± 1 ^a^
Neutrophils (%)	44.86 ± 34.91	25.50 ± 22.57	29.67 ± 20.13	26.17 ± 6.18
Eosinophils (%)	0.71 ± 0.49	0.67 ± 0.82	0.67 ± 0.52	0.67 ± 0.52

Values are means ± SD. NX: normoxia; NX+CH: normoxia plus *S. nutans*; AHH: acute hypobaric hypoxia; AHH+CH: acute hypobaric hypoxia plus *S. nutans*; Mean corpuscular volume (MCV); Mean corpuscular hemoglobin concentration (MCHC); Red blood cells (RBC). ^a^ *p* < 0.05 vs. NX (48 h), ^b^ *p* < 0.05 vs. AHH (48 h), ^c^ *p* < 0.05 vs. NX+CH (48 h).

**Table 3:** Primer sequences used in gene expression analysis.

Gen	Forward	Reverse	Tm	Amplicon Length	GC%	Amplification Efficiency (%)	R^2^
HIF-1α	CCTAACAGTCCCAGTGAGTA	TTCTTCGCTTCTGTGTCTTC	60°	107	50	98%	0.9964
TNF-α	CGTGTTCATCCGTTCTCTAC	GAGCCACAATTCCCTTTCT	60°	127	50	100%	0.9738
IL-1β	GAACCCGTGTCTTCCTAAAG	GACTTGGCAGAGGACAAAG	60°	120	50	100%	0.9883
IL-6	AAAGCCAGAGTCATTCAGAG	CTCCATTAGGAGAGCATTGG	60°	117	45	100%	0.9853
NF-kB	GGATCCAGTGTGTGAAGAAG	GACCGCATTCAAGTCATAGT	60°	122	50	96%	0.9944
VEGF	CGAGGCAGCTTGAGTTAAA	CTTTCCGGTGAGAGGTCTA	60°	116	47	97%	0.9713
β-Actina	GACCTGACAGACTACCTCAT	GAAGTCTAGGGCAACATAGC	60°	120	50	99%	0.9768
GAPDH	CTCCCATTCTTCCACCTTTG	TGGTCCAGGGTTTCTTACT	60°	120	50	96%	0.9898

## Data Availability

The original contributions presented in this study are included in the article. Further inquiries can be directed to the corresponding authors.

## Abbreviations

The following abbreviations are used in this manuscript:

AHH	Acute Hypobaric Hypoxia
AMS	Acute Mountain Sickness
HAPE	High Altitude Pulmonary Hypertension
CH	Chachacoma
NX	Normobaric normoxia
NX+CH	Normobaric normoxia plus *S. nutans* administration
AHH+CH	Acute Hypobaric Hypoxia plus *S. nutans* administration
HIF-1α	Hypoxia-inducible factor-1α
NF-κB	Nuclear factor kappa-light-chain-enhancer of activated B cells
TNF-α	Tumor necrosis factor alpha
IL-1β	Interleukin-1β
IL-6	Interleukin-6
VEGF	Vascular Endothelial Growth Factor
MDA	Malondialdehyde
MCV	Mean corpuscular volume
MCHC	Mean corpuscular hemoglobin concentration
RBC	Red blood cell
Hct	Hematocrit
Hb	Hemoglobin
